# Dexmedetomidine suppresses microglial activation in postoperative cognitive dysfunction via the mmu-miRNA-125/TRAF6 signaling axis

**DOI:** 10.1515/med-2025-1236

**Published:** 2025-07-23

**Authors:** Zhiyan Xu, Kaihua Zhong, Weiyuan Chen, Huixia Lan, Weifeng Zhong, XiaoHong Wang, Mu Chen, Bin Pan

**Affiliations:** Meizhou People’s Hospital (Meizhou Academy of Medical Sciences), Meizhou, China; Department of Urology, Guangzhou Twelfth People’s Hospital, Guangzhou, 510620, China; Department of Nephrology, Third Affiliated Hospital of Southern Medical University, Guangzhou, 510630, China; Department of Respiratory Medicine, Guangzhou Eighth People’s Hospital, Guangzhou, 510060, China; Department of Urology, The First Affiliated Hospital of Jinan University, Guangzhou, 510630, China; Guangdong Provincial Key Laboratory of Precision Medicine and Clinical Translational Research of Hakka Population, Meizhou, 514021, China

**Keywords:** POCD, dexmedetomidine, miR-125a, TRAF6, microglial activation

## Abstract

**Background:**

Postoperative cognitive dysfunction (POCD) is driven in part by microglial activation and the resulting neuroinflammatory response. Emerging evidence suggests that microRNAs regulate key inflammatory pathways in the central nervous system. In this study, we examined the role of the mmu‑miR‑125a/TRAF6 signaling axis in microglial activation under inflammatory conditions induced by lipopolysaccharide (LPS) and surgical trauma and evaluated whether dexmedetomidine (DEX) modulates this pathway to alleviate POCD.

**Methods:**

Murine microglial cells were treated with LPS to induce activation. Expression levels of mmu‑miR‑125a and TRAF6 were quantified by qRT‑PCR and Western blotting. Bioinformatic prediction of miRNA binding sites was performed, and a luciferase reporter assay was used to confirm direct targeting of TRAF6 by mmu‑miR‑125a. Adult mice underwent standardized surgical trauma to induce POCD. Brain tissues were analyzed for microglial activation markers, cytokine levels, and expression of mmu‑miR‑125a and TRAF6. DEX was administered in both *in vitro* and *in vivo* models. The effects on cytokine release, microglial activation, and the mmu‑miR‑125a/TRAF6 axis were assessed.

**Results:**

Our findings revealed significant alterations in the expression levels of TRAF6 and mmu-miR-125a during LPS-induced microglial activation. Through bioinformatics analysis and experimental validation, we identified TRAF6 as a direct target of mmu-miR-125a. The mmu-miR-125a/TRAF6 axis was found to be crucial for regulating microglial activation both *in vitro*, using an LPS-induced model, and *in vivo,* using a surgical trauma-induced POCD model. Moreover, we demonstrated that DEX, an alpha-2 adrenergic receptor agonist, effectively modulated the inflammatory cytokine release by targeting the mmu-miR-125a/TRAF6 axis in both models. The administration of DEX significantly suppressed microglial activation and TRAF6 expression, effects that were reversed by the inhibition of mmu-miR-125a.

**Conclusion:**

Our study provides new insights into the molecular mechanisms underlying microglial activation and highlights the therapeutic potential of targeting the mmu-miR-125a/TRAF6 axis to alleviate neuroinflammation by the administration of DEX in POCD.

## 1 Introduction

Elderly patients are highly susceptible to brain function disorders following surgery, which is collectively referred to as postoperative cognitive dysfunction (POCD) [1]. POCD was first identified in elderly patients following cardiac surgery, but recent studies have shown a high incidence in patients undergoing other types of surgeries as well [2–4]. Reports indicate that the incidence of POCD 7 days after non-cardiac surgery in patients over 60 years old is 25.8%, and the incidence 3 months postoperatively is approximately 9.9% [5]. In contrast, the overall incidence of POCD after cardiac surgery can reach 51% [6]. POCD leads to delayed patient recovery, increased complications, loss of self-care ability, prolonged hospitalization, increased medical costs, and a series of medical, social, and economic issues. The exact pathogenesis of POCD remains unclear; it is often the result of multiple factors acting together and is directly associated with vascular damage and inflammation, with the extent of damage positively correlated with vascular and inflammatory responses [7]. The immune response triggered by surgery is most likely the “trigger point” causing POCD. The inflammatory response plays a crucial role in the occurrence and development of POCD [8,9]. Neuroinflammation is a significant factor leading to cognitive impairment and is also closely related to neurodegenerative diseases in the elderly, such as Alzheimer’s disease (AD), Parkinson’s disease (PD), Huntington’s disease, multiple sclerosis (MS), and amyotrophic lateral sclerosis [10,11].

TRAF6 (TNF receptor-associated factor 6), a member of the TRAF family of proteins, was first reported to be involved in inflammatory signaling pathways [12]. TRAF6 acts as an adaptor protein, mediating signals induced by the TNF receptor family, including TNFR. TRAF6 recognizes and binds to signals such as CD40 on receptor cells, subsequently activating NF-kB and MAPK, playing a crucial role in immune responses [13]. Studies have shown that lipopolysaccharide (LPS)-induced activation of dendritic cells leads to the production of TNF-alpha and IL-13, a process mediated by the TLR4/MyD88/TRAF6 signaling pathway [14]. TRAF6 can activate TNF-alpha, which in turn induces the production of inflammatory factors such as IL-6 and IL-8. Pharmacological interventions targeting the function of TRAF6 can modulate local inflammation [15]. Therefore, regulating TRAF6 can, to some extent, control the inflammatory process; however, the role and the regulatory mechanism of TRAF6-involved inflammatory response in POCD remain unclear.

In recent years, numerous international reports have indicated that dexmedetomidine (DEX) has a certain interventional effect on cognitive dysfunction in elderly patients after general anesthesia [16]. Data confirm that the incidence of delirium and coma observed with DEX use is significantly lower than that in the control group, and survival time is prolonged, with only 6% of the general anesthesia patients experiencing postoperative delirium [17]. In contrast, the incidence of postoperative delirium is as high as 45% when general anesthesia is administered with drugs such as propofol or midazolam [18]. DEX is a new generation of highly selective α2-adrenergic receptor agonists, which exerts dose-dependent sedative and hypnotic effects, similar to the action of remifentanil during anesthesia [19]. It also exerts analgesic and sympatholytic effects by activating α2 receptors located in the central nervous system and peripherally, thereby inhibiting the stress response [20]. Recently, DEX has also been found to have anti-inflammatory effects [21,22], although the mechanism of these effects remains unknown.

To elucidate the mechanism by which DEX regulates POCD, this study aims to further investigate through *in vitro* experiments, as well as an *in vivo* mouse model of POCD induced by surgical anesthesia, to determine the effects of TRAF6-mediated neuroinflammation.

## 2 Methods and materials

### 2.1 Animals and ethics

BALB/c mice were obtained from the Guangdong Medical Laboratory Animal Center in Guangzhou, China. They were kept under specific pathogen-free (SPF) conditions, with a 12-h light/dark cycle in rooms where temperature and humidity were controlled. The mouse was allowed to acclimate to their new environment for 1 week before any experiments began. All procedures involving the animals followed the NIH Guide for the Care and Use of Laboratory Animals and received approval from Shantou University.

### 2.2 Cell culture and transfection

BV-2 murine microglial cell lines, obtained from the Cell Bank of the Chinese Academy of Sciences (Shanghai, China), were cultured in DMEM/F12 medium with 10% FBS in a humidified atmosphere at 37°C with 5% CO_2_. The cells were transfected with mmu-miR-125a mimics, inhibitors (50 nM), or negative controls (Genepharma Biotechnology, Shanghai, China) using Lipofectamine 2000 (Invitrogen, Carlsbad, CA, USA). For primary microglial cell culturing, cells were isolated from the brains of 1-day-old SD BALB/c mice pups, following a previously established method [23]. After 10 days, the mixed culture, which included microglia, astrocytes, and oligodendrocytes, was centrifuged at 220 rpm for 2 h. The supernatants were transferred to new cell culture plates, and non-adherent cells, mainly astrocytes and oligodendrocytes, were removed after 15 min. Then, the cells were cultured and treated as BV2 cells. For LPS treatment, LPS (InvivoGen, Hong Kong, China; cat no. #tlrl-eblps, final concentration 50 or 100 ng/mL diluted in culture medium) was added to the cell culture for 24 h. For DEX (Selleckchem, CA, USA, cat no. #S3075) administration, a final concentration of 1 μM in DMSO was added prior to the addition of LPS.

### 2.3 RT-qPCR

Total RNA was extracted with TRIzol (Invitrogen, Carlsbad, CA, USA) as per the manufacturer’s instructions and reverse-transcribed with a cDNA synthesis kit (Thermo Fisher Scientific). After that, the SYBR premix ExTaq II kit (TaKaRa, Dalian, China) was used to perform the RT-qPCR according to the manufacturer’s procedure. Relative mmu-miR-125a and TRAF6 mRNA expression values were normalized to U6 and GAPDH using the delta-delta-Ct method. The primers were TRAF6 forward, 5′-TTTCCCTGACGGTAAAGTGCCC-3′, TRAF6 reverse, 5′-ACCTGGCACTTCTGGAAAGGAC-3′; mmu-miR-125a forward, 5′-CCCTGAGACCCTTTAACC-3′, mmu-miR-125a reverse, 5′-GAACATGTCTGCGTATCTC-3′.

### 2.4 Western blotting

Protein concentration was measured using the BCA protein concentration detection kit from Aspen (Wuhan, China). Equal amounts of protein were separated on 10% SDS-PAGE and transferred to a PVDF membrane. The membrane was incubated overnight at 4°C with the following primary antibodies: anti-TRAF6 (66498-1-Ig, 1:1,000; Proteintech, Rosemont, IL, USA) and GAPDH (60004-1-Ig, 1:10,000; Proteintech). The membrane was then incubated with a secondary antibody (A0216, 1:5,000; Beyotime, Shanghai, China) at room temperature. Bands were visualized using an enhanced chemiluminescence detection kit, and their intensities were analyzed.

### 2.5 Dual luciferase reporter assay

Online tools such as TargetScan [24] were used to search for the potential binding site between mmu-miR-125 and TRAF6. Then, the 3′-UTR sequence of TRAF6, either containing the predicted miR-125 binding site (WT) or a mutated version (MUT), as indicated in Figure 2a, was synthesized by GenePharm (Shanghai, China) and inserted into the pmirGLO vector (Promega, Madison, WI, USA). Cells were then co-transfected with a negative control and a mmu-miR-125a mimic. After 48 h, a dual-luciferase reporter assay was performed following the manufacturer’s instructions.

### 2.6 Immunofluorescence assay

Mice were anesthetized and then underwent cardiac perfusion with PBS, followed by a 4% paraformaldehyde (PFA) solution for fixation. Then, the tissues were dehydrated, embedded, and slices were prepared at a thickness of 20 μm and stored at −80°C for preservation. For immunofluorescence staining, the frozen brain sections were washed three times with PBS, blocked with 10% goat serum at 37°C for 1 h, and incubated overnight at 4°C with an anti-Iba-1 antibody (1:500, Cell Signaling Technology, USA). The sections were then incubated with Alexa Fluor 488-conjugated goat anti-rabbit IgG (1:500, Beyotime, Shanghai, China) at 37°C for 1 h. Following staining with DAPI in an anti-fluorescence quenching solution, images were captured using a laser scanning confocal microscope (ZEISS, Oberkochen, Germany).

### 2.7 ELISA

To measure the expression levels of TNF-α, IL-6, and IL-1β in BV2 cell lysates or serum levels of surgical treated mice following treatments, supernatants from these samples were analyzed using an ELISA kit according to the manufacturer’s instructions (R&D Biosciences, San Diego, CA, USA).

### 2.8 Animal POCD model

The commonly used surgical trauma model of POCD was implemented following established protocols [23]. First, the mice were anesthetized using halothane [29], and a longitudinal incision of 6 cm was made along the dorsal midline and another 5 cm along the abdominal midline. The intestines were then exteriorized and left exposed for 5 min. To prevent infection, polysporin (Pfizer, Markham, Ontario, Canada) was applied to the surgical site. In the sham-operated group, mice were also anesthetized, and their abdominal and dorsal regions were shaved and cleaned, but no incisions were made. Both groups were kept under halothane anesthesia for the same duration. A concentration of 50 μg/kg of Dex was selected for this model accordingly [25].

### 2.9 Open field test

The open field test was applied as previously reported [26,27]. Briefly, each group included at least three BALB/c mice, and mice were placed in an open field arena for a duration of 10 min. Seven days post-surgery, they were returned to the arena. Using ANY-maze tracking software, the duration spent by the mice in the corner, border, and center areas, as well as their overall travel distance, was recorded and measured.

### 2.10 Statistical analysis

Data are presented as mean ± standard error from at least three independent experiments and were analyzed using SPSS software version 21.0 (SPSS Inc., Chicago, IL, USA). Statistical analysis involved analysis of variance (ANOVA) followed by the Bonferroni post hoc test for multiple comparisons. For single comparisons between two groups, an unpaired Student’s *t*-test was used. A *P*-value of less than 0.05 was considered statistically significant.

## 3 Results

### 3.1 TRAF6 is upregulated while mmu-miR-125a is downregulated during LPS-induced microglial activation

**Figure 1:**
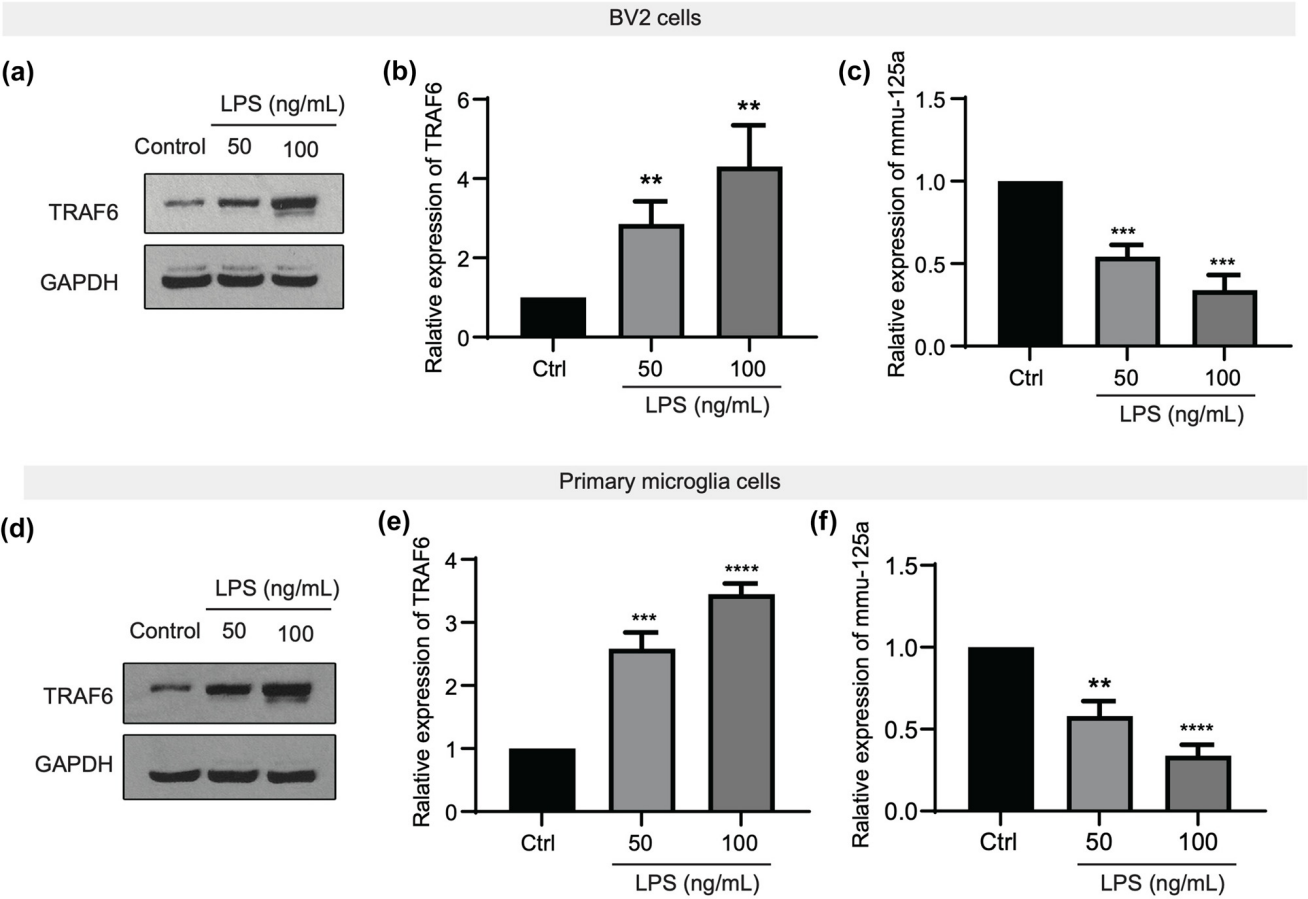
TRAF6 is upregulated while mmu-miR-125a is downregulated during LPS-induced microglial activation. (a)–(c) BV2 cells were cultured and treated with LPS at the indicated concentration. Then, the cells were subjected to western blotting to detect the level of TRAF6 (a) and (b) and mmu-miR-125a (c). (d)–(f) Primarily cultured microglial cells were treated with LPS, and the cells were harvested for the detection of the TRAF6 protein level (d) and (e) with western blotting and RT-qPCR of mmu-miR-125a. ANOVA, **P < 0.01; ***P < 0.001; ****P < 0.0001.

To explore the role of TRAF6 and mmu-miR-125a during the inflammatory response, we applied a commonly used cellular model with LPS administration [23]. BV2 cells and primarily cultured microglia cells were treated with 50 or 100 ng/mL LPS. Then, cells were harvested and subjected to western blotting or RT-qPCR assays. As shown in Figure 1a and b, compared to the control group, the treatment of LPS significantly induced the upregulation of the TRAF6 protein level in BV2 cells. Moreover, the mmu-miR-125b expression, revealed by RT-qPCR, was markedly downregulated under the treatment of LPS (Figure 1c). The upregulation of TRAF6 and the downregulation of mmu-miR-125b were also observed in cultured primary microglia cells, showing a negative relationship between the two (Figure 1d–f). These data suggest that TRAF6 and mmu-miR-125a were involved in LPS-induced microglial activation.

### 3.2 TRAF6 is a direct target of mmu-miR-125a

**Figure 2:**
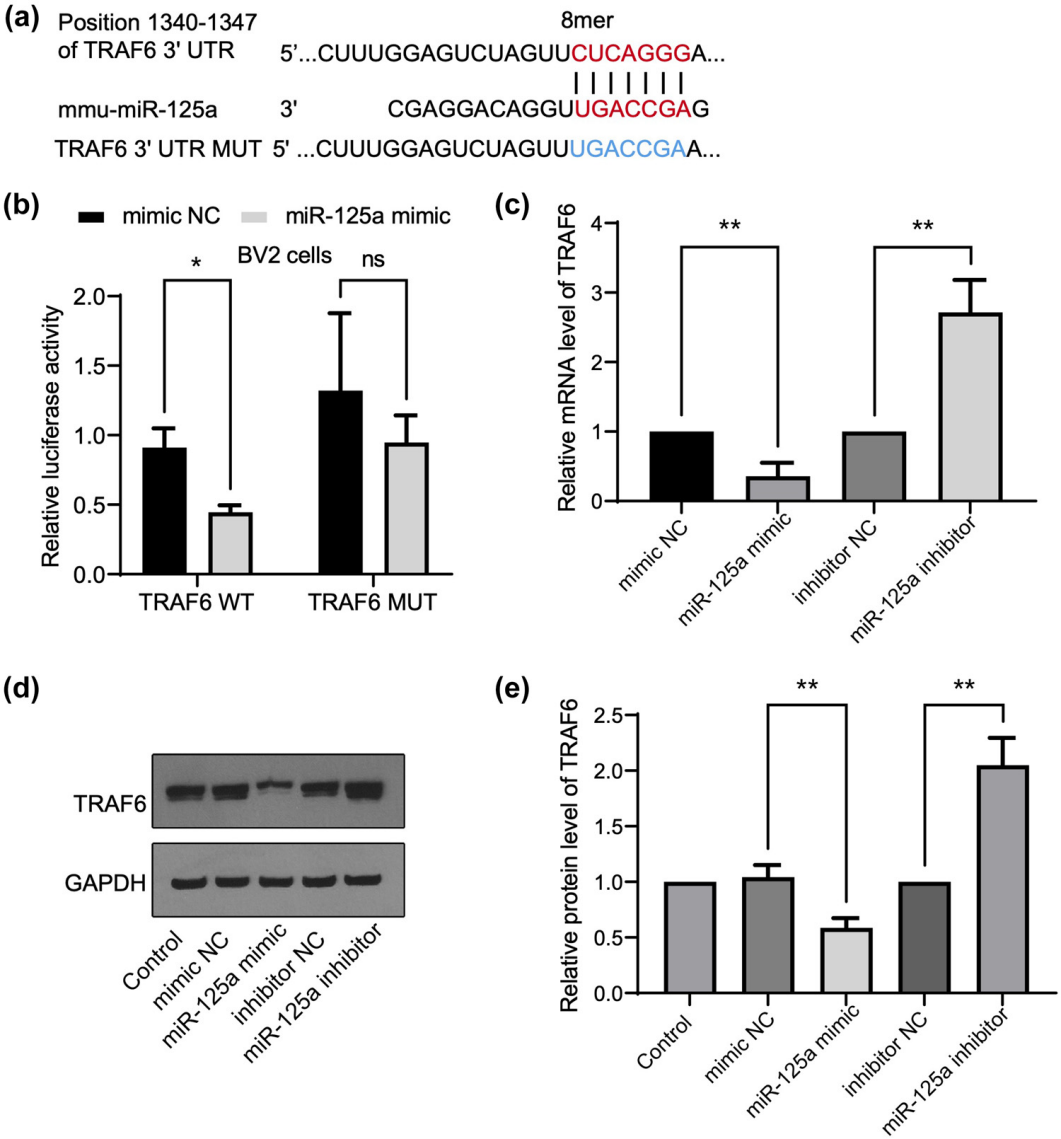
TRAF6 is a direct target of mmu-miR-125a. (a) The sequence of the TRAF6 3′ UTR region matched with mmu-miR-125a and the constructed mutant. (b) BV2 cells were transfected with TRAF6 wild type (WT) and mutant (MUT) luciferase reporter plasmids, together with mimic NC or mmu-miR-125a mimics. The relative luciferase activity is shown. (c) The mRNA level by RT-qPCR was detected by the transfection of the indicated miR-125a fragments or the controls. (d) and (e) The protein level of TRAF6 was detected by western blotting under the treatment as in (c). Student’s *t*-test between two groups, **P < 0.01.

To further investigate the regulatory mechanism of TRAF6 in the inflammatory response, we utilized bioinformatics tools such as TargetScan [24] to identify potential miRNAs that target TRAF6. As illustrated in Figure 2a, bioinformatics analysis of the mouse database identified that the sequence of mmu-miR-125a matched with the 3′-UTR region of TRAF6, suggesting a potential regulatory relationship. To investigate this further, the matched sequence of the WT 3′-UTR and a mutated clone (MUT, as shown in Figure 1a) was cloned into a luciferase reporter vector for subsequent dual luciferase reporter assays. Figure 2b shows that, compared to scrambled mimic NC (the control group), transfection of the mmu-miR-125a mimic significantly reduced the luciferase activity of the TRAF6 WT reporter in BV2 cells, while it did not affect the TRAF6 MUT reporter. Additionally, transfection with mmu-miR-125a mimic (compared to the mimic NC group) significantly decreased the mRNA (Figure 2c) and protein levels (Figure 2d and e) of TRAF6, whereas transfection with the mmu-miR-125a inhibitor (compared to the inhibitor of the NC group) increased these levels. These results indicate that TRAF6 is a direct target of mmu-miR-125a in BV2 cells.

### 3.3 DEX attenuates LPS-induced microglia activation via modulating the mmu-miR-125a/TRAF6 axis

**Figure 3:**
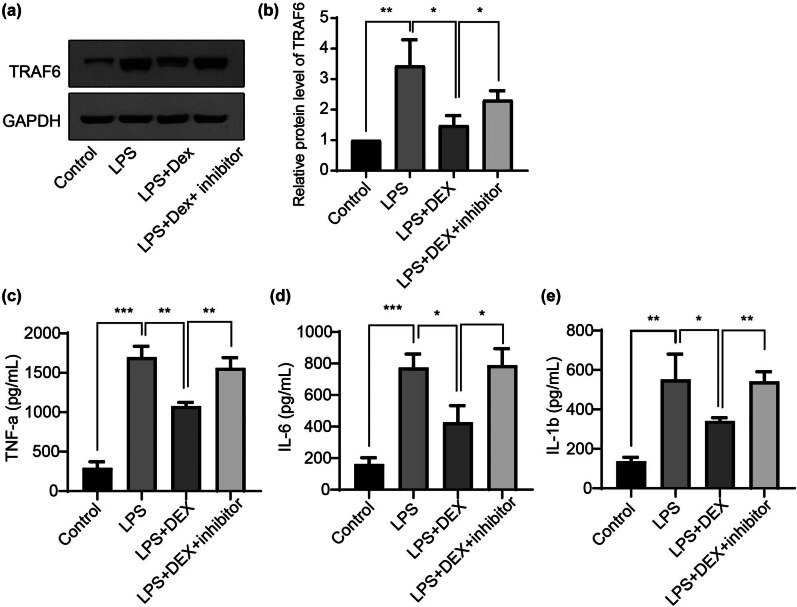
Dexmedetomidine attenuates LPS-induced microglia activation via modulating the mmu-miR-125a/TRAF6 axis. (a) and (b) BV2 cells were treated with LPS and DEX or miR-125a inhibitor, then the cell lysates were subjected to western blotting for determining the TRAF6 level (fold changes). The supernatant from the same treatment was subjected to an ELISA assay to detect the secreted levels of TNF-alpha (c), IL-6 (d), and IL-1beta (e). ANOVA multiple comparisons, *P < 0.05; **P < 0.01; ***P < 0.001.

Previous reports showed that DEX revealed the protective role in neuronal dysfunction [28] and microglial activation [29,30]; however, whether DEX functions via the mmu-miR-125a/TRAF6 axis remains to be further explored. As shown in Figure 3a and b, compared to the PBS control, LPS administration in BV2 cells significantly induced the upregulation of TRAF6, which could be counteracted by the treatment of DEX (group 3). However, this impact of DEX could be further inhibited by the transfection of the mmu-miR-125a inhibitor, inducing the re-upregulation of the TRAF6 level. We further explored the changes in the inflammatory response indicators in the culture media upon these treatments. By the application of ELISA assay, LPS treatment-induced upregulation of TNF-alpha (Figure 3c), IL-6 (Figure 3d), and IL-1beta (Figure 3e) could be suppressed by the treatment with DEX, but further restored by the transfection of the mmu-miR-125a inhibitor. These data suggest that DEX attenuates LPS-induced inflammatory response via modulating the mmu-miR-125a/TRAF6 axis.

### 3.4 DEX alleviates neuroinflammation in a POCD surgical trauma model via the mmu-miR-125a/TRAF6 axis

**Figure 4:**
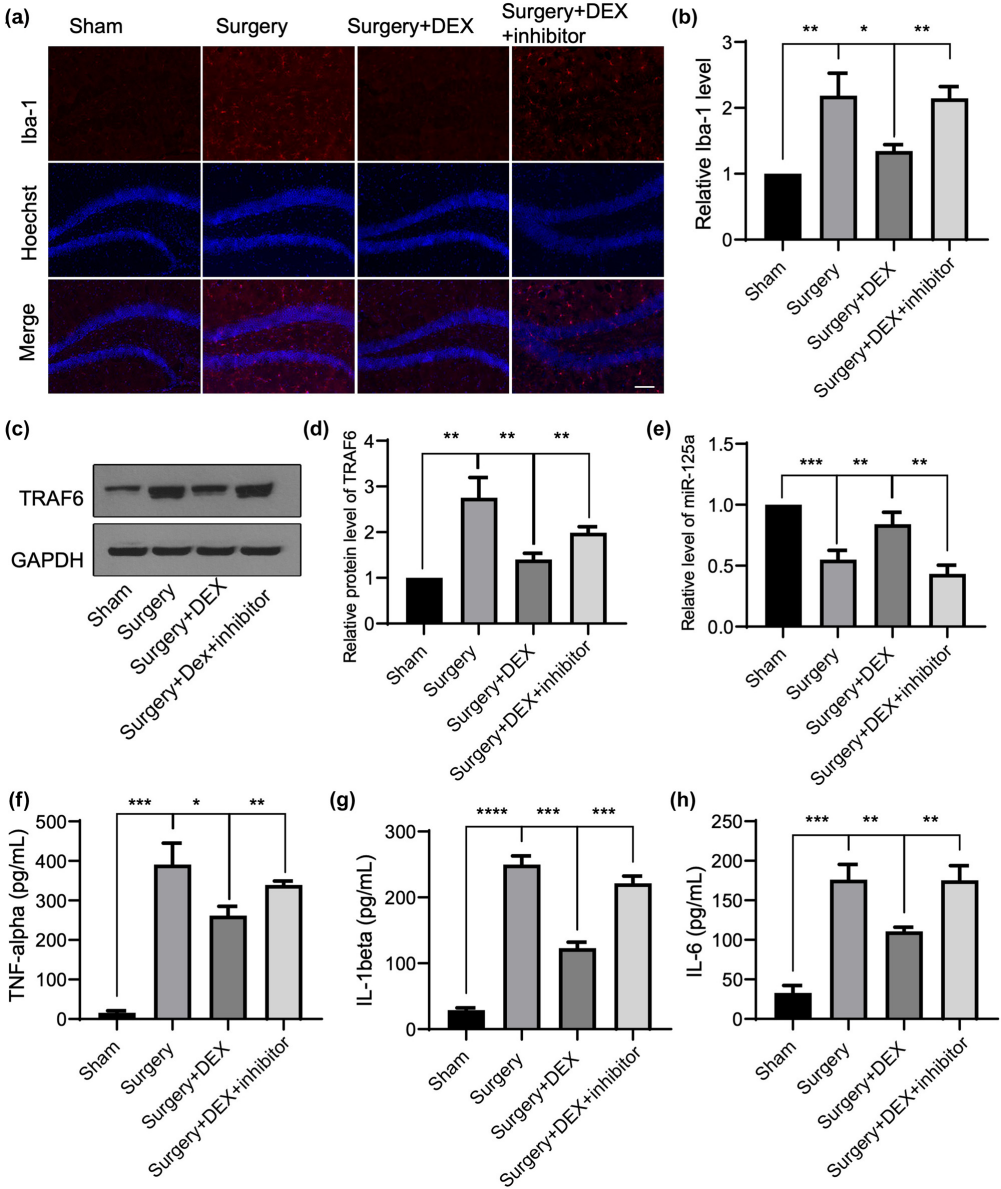
Dexmedetomidine alleviates neuroinflammation in a POCD surgical trauma model via the mmu-miR-125a/TRAF6 axis. (a) and (b) A POCD surgical trauma model was established, and then mice were treated with Dex or injected with the mmu-miR-125a inhibitor. The Sham group was used as the control group of the surgery group. After surgery or pharmacal administration, the hippocampus tissues were subjected to immunohistochemistry to detect the level of Iba-1 (red, for activated microglia). (c) and (d) The protein level of TRAF6 and mmu-miR-125a (e) in the hippocampus was detected and quantified as relative fold changes. The serum level from treated mice was collected to detect the level of TNF-alpha (f), IL-6 (g), and IL-1beta (h). Scale bar, 20 μm. ANOVA multiple comparisons, *P < 0.05; **P < 0.01; ***P < 0.001; ****P < 0.0001.

**Figure 5:**
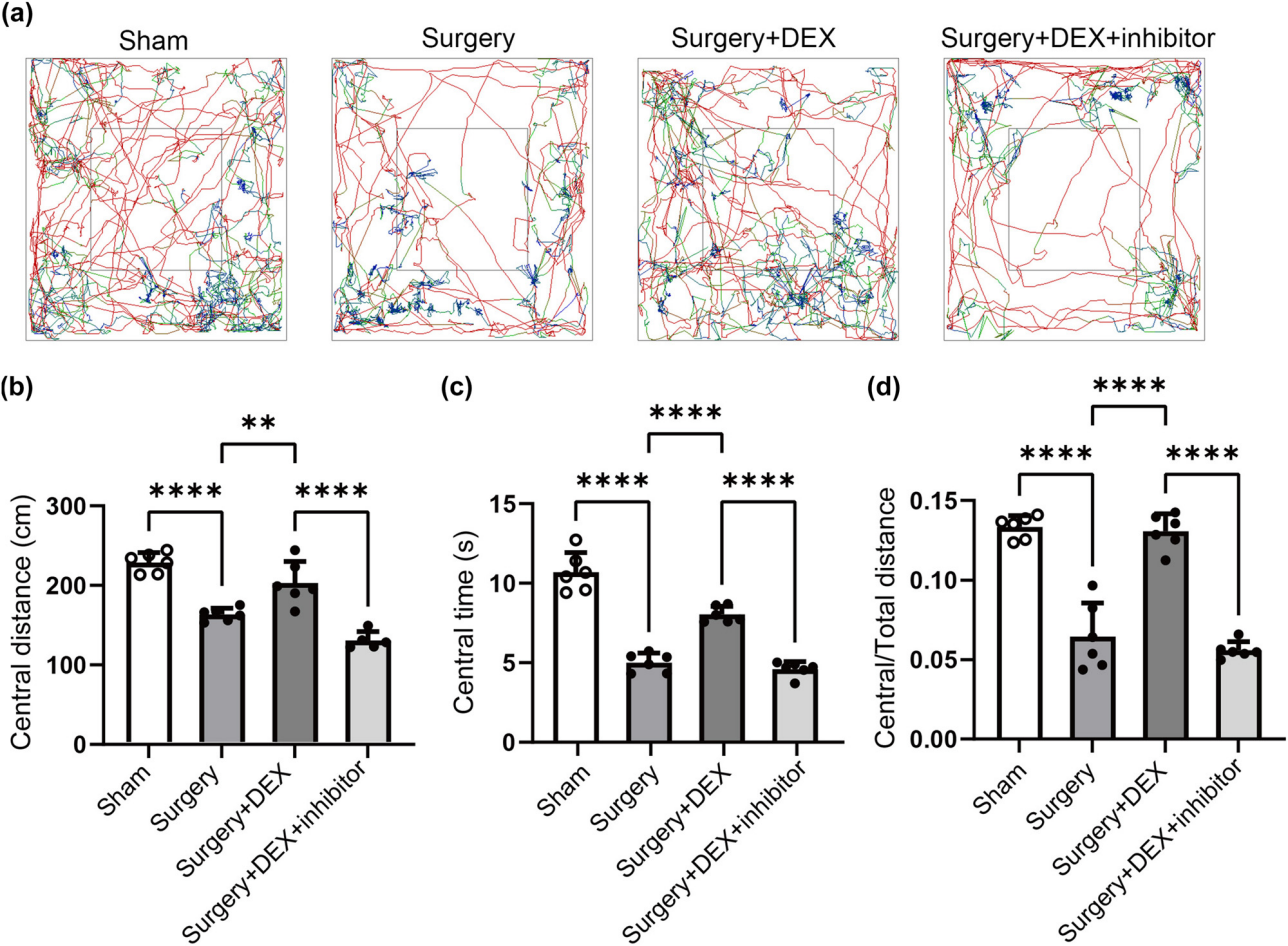
The open field test. Mice were treated as in Figure 4, and then subjected to an open field test. Then, the central distance, central time, and central vs total distance were collected and calculated (*n* = 6). (a) The representative images in each group are shown. (b)–(d) Statistical data. ANOVA multiple comparisons; *P < 0.05; **P < 0.01; ***P < 0.001; ****P < 0.0001.

To further determine the effect of DEX on neuroinflammation *in vivo*, we established a mouse model of surgical trauma, as previously reported [23,31]. As shown in Figure 4a and b, the immunofluorescence staining of Iba-1 revealed that, compared to the Sham control group, the surgical model significantly induced microglial activation in the mouse hippocampus. The administration of DEX markedly suppressed this microglial activation, an effect that was counteracted by the injection of a mmu-miR-125a inhibitor (Figure 4a and b). Western blot analysis demonstrated that the protein level of TRAF6 increased following surgery compared to the Sham group, but this increase was suppressed by DEX administration (Figure 4c and d). The injection of the mmu-miR-125a inhibitor reversed the downregulation of TRAF6. RT-qPCR revealed that the endogenous level of mmu-miR-125a was reduced following the POCD surgical model, but it could be upregulated by DEX administration and suppressed by the co-injection of a mmu-miR-125a inhibitor (Figure 4e). To further illustrate the impact of DEX, animals were subjected to an open field test to determine the locomotion, exploratory activity, and anxiety-like behavior changes in POCD models [26,27]. As shown in Figure 5, surgical-induced POCD mice spent less time in the central area and showed reduced central distance. Application of DEX significantly alleviates these changes, with increased central time and distance, which could again be abolished by the administration of the miR-125a inhibitor (Figure 5c and d). These data suggest that DEX alleviates neuroinflammation in this POCD surgical trauma model via regulating the mmu-miR-125a/TRAF6 axis.

## 4 Discussion

In the present study, we demonstrated that the expression levels of TRAF6 and mmu-miR-125a were significantly altered during LPS-induced microglial activation and inflammatory response. TRAF6 was identified as a direct target of mmu-miR-125a, and the mmu-miR-125a/TRAF6 axis played a crucial role in microglial activation in both the LPS-induced model *in vitro* and the surgical trauma-induced POCD model *in vivo*. Additionally, DEX regulated inflammatory cytokine release by modulating the mmu-miR-125a/TRAF6 axis in both settings. Targeting the miR-125a/TRAF6 axis may alleviate microglial activation-related neuroinflammation and offer a potential therapeutic strategy for the clinical treatment of POCD.

POCD is a common complication following surgery, particularly affecting elderly patients [32]. It is characterized by a decline in cognitive function, including memory and learning abilities [33]. Recent research has focused on the role of microRNAs (miRNAs) in the pathogenesis and potential treatment of POCD. For example, miR-190a-3p is significantly associated with POCD and regulates the gene Tiam1, which is involved in pathways related to psychiatric disorders and brain development [34]. miR-572 is downregulated in POCD and regulates the expression of NCAM1, aiding in the restoration of cognitive function [35]. miR-181b-5p attenuates POCD by reducing hippocampal neuroinflammation and targeting proinflammatory mediators like TNF-α [36]. Also, the circRNA-associated-ceRNA network is involved in the regulation of POCD, such as CircRNA-associated-ceRNA networks, involving miRNAs such as mmu-miR-298-5P, play a role in POCD by modulating pathways like Wnt and VEGF signaling [37,38]. Moreover, lncRNAs’ interactions with miRNA are involved in POCD; for example, lncRNA NONMMUT055714 acts as a sponge for miR-7684-5p, protecting against POCD by regulating SORLA and reducing neuroinflammation and neuronal apoptosis [39,40]. For potential clinical applications, preoperative serum levels of miR-155 can independently predict the occurrence of POCD after surgery [41]. Thus, the relationship between miRNAs and POCD is multifaceted, involving the regulation of genes and pathways associated with neuroinflammation, neuronal apoptosis, and cognitive function. MiRNAs play crucial roles in the development and potential treatment of POCD. Although the role of miR-125a in inflammation has been widely reported [42,43], the impact of miR-125a in POCD remains to be explored. Here, we found that mmu-miR-125a was involved in LPS-induced inflammatory response and surgery trauma-induced POCD mice. The results are consistent with the above observations.

TRAF6 is a critical adaptor protein involved in the regulation of inflammatory responses [44]. It plays a significant role in various signaling pathways, particularly those mediated by Toll-like receptors (TLRs) and cytokine receptors [45]. Understanding the relationship between TRAF6 and the inflammatory response is essential for developing therapeutic strategies to manage inflammation-related diseases. TRAF6 is crucial for TLR-mediated signaling, leading to the production of pro-inflammatory cytokines [46]. TRAF6 is essential for maintaining immune homeostasis by regulating T-cell responses and preventing excessive inflammation [47]. TRAF6 is involved in the production of inflammatory cytokines through its interaction with various signaling molecules, such as GSK3β and NF-κB [48,49]. TRAF6 phosphorylation by MST4 kinase suppresses its oligomerization and autoubiquitination, thereby reducing inflammation [50]. Moreover, TRAF6 contributes to chronic inflammatory pain by regulating microglial activation in the spinal cord [51].

On the other hand, TRAF6 is an E3 ubiquitin ligase that activates NF-κB and MAPK pathways, which may contribute to the regulation of POCD, for example, by mediating K63-linked polyubiquitination of signaling intermediates. In neuroinflammation following traumatic brain injury (TBI), TRAF6 drives NF-κB and MAPK activation in astrocytes, increasing pro-inflammatory chemokines like CCL2 and CXCL10. Inhibition of TRAF6 via RNAi or pharmacological blockers (e.g., MAPK/NF-κB inhibitors) reduces neuroinflammation and secondary neuronal damage [52]. TRAF6’s K63-linked ubiquitination suppresses NF-κB/MAPK activation and reduces cytokine production in macrophages [53]. Also, TRIM60-mediated SUMOylation of TAB2 (a TRAF6-binding partner) suppresses TRAF6–TAK1 complex formation, inhibiting MAPK/NF-κB signaling in LPS-induced inflammation [54]. Enhanced TRAF6 signaling in astrocytes activates JNK and NF-κB through K63 ubiquitination, inhibits autophagy, and reduces neuroprotective effects, leading to decreased ROS clearance capacity and neuronal damage [55]. As to POCD, miR-146a is reported to be associated with cognitive decline by suppressing hippocampal neuroinflammation in mice, via regulation of the IRAK1/TRAF6/NF-κB pathway [56]. Here, our data indicated that TRAF6 was upregulated in LPS-induced inflammatory response and the surgical trauma POCD mice model. DEX treatment suppressed the protein level of TRAF6 and the subsequent inflammation. Targeting TRAF6 and its regulatory mechanisms offers promising therapeutic potential for managing neuroinflammatory conditions.

DEX has been investigated for its potential ability to mitigate POCD. DEX administration can improve cognitive scores, such as Mini-Mental State Examination (MMSE) and Montreal Cognitive Assessment (MoCA) scores at 24 and 72 h post-operation [18]. Meta-analyses and clinical trials indicate DEX use is associated with a lower incidence of POCD on the first and seventh postoperative days, with higher MMSE scores compared to control groups [57,58]. Intraoperative DEX infusion reduced the incidence of POCD compared to esmolol in patients undergoing middle ear surgery under hypotensive anesthesia [59]. DEX treatment reduces inflammatory factors, such as serum levels of S100β, neuron-specific enolase (NSE), norepinephrine, cortisol, interleukin-6 (IL-6), and tumor necrosis factor-alpha (TNF-α) in POCD patients [57]. DEX reduced hippocampal neuron apoptosis, neuroinflammation, and cognitive impairment in aged mice, suggesting a protective role against POCD via the miR-381/EGR1/p53 signaling pathway [60]. However, some studies showed that there is no significant difference in postoperative delirium and cognitive performance between DEX and placebo groups [61,62], highlighting the importance of timing and context in DEX administration. Thus, the effect of DEX application in the treatment of POCD remains to be further explored. Consistent with the previous positive reports, our data suggest that DEX administration significantly suppressed the inflammatory response in both the LPS cell model and surgical trauma mice model, via targeting the mmu-miR-125a/TRAF6 axis.

In general, our findings demonstrated that the involvement of TRAF6 and mmu-miR-125a during LPS-induced microglial activation and inflammatory response. TRAF6 is a direct target of mmu-miR-125a during the processes, and DEX alleviates inflammation via modulating the mmu-miR-125a/TRAF6 axis in both *in vitro* and *in vivo* models. DEX shows promise in reducing the severity of POCD through its neuroprotective and anti-inflammatory effects.
